# A Simplified HPLC Method for Quantification of Torsemide from Human Plasma and its Application to a Bioequivalence Study

**DOI:** 10.4103/0250-474X.44609

**Published:** 2008

**Authors:** I. J. Khan, P. Loya, M. N. Saraf

**Affiliations:** Department of Pharmacology, The Bombay College of Pharmacy, Kalina, Santacruz (E), Mumbai-400 098, India

**Keywords:** Torsemide, HPLC, bioequivalence

## Abstract

A simple, rapid and selective method was developed. The method was validated and found to be linear in the range of 100-4000 ng/ml. Chromatographic peaks were separated by means of a 5 μm, C18 silica column using acetonitrile and phosphate buffer (0.05 M) in proportion of 40:60 (pH 4.0) as a mobile phase. The retention time of torsemide was 5.00±0.20 min. The chromatograms showed good resolution and no interference from plasma. The mean recovery from human plasma was found to be above 82%. Both inter-day and intra-day accuracy and precision data showed good reproducibility. This method was applied to a single dose bioequivalence study. Log transformed values were compared by ANOVA followed by classical 90% confidence interval. Confidence limits for C_max_, AUC_0-t_ and AUC_0-inf_ ranged from 98.6 to 102.8, 101.8 to 105.3 and 102.4 to 105.5 respectively. These results suggested that the analytical method was linear, precise and accurate. Test and reference product were found to be bioequivalent.

Torsemide [1-isopropyl-3-[(4-*m*-toluidino-3-pyridyl)] sulfonylurea, a loop diuretic. Following oral administration of torsemide, peak concentration is usually achieved within 1-2 h with an elimination half-life of approximately 3.5 h (http://www.rxlist.com accessed on Aug 2005). Literature survey revealed various methods for the estimation of torsemide from human plasma 1–8. Reported methods involve complicated time-consuming multi-step liquid-liquid extraction techniques. The present paper reports on the development of a rapid, sensitive and validated HPLC method for quantification of torsemide from human plasma after single step treatment of the plasma sample. The developed and validated method was successfully applied to a pharmacokinetic study in healthy human volunteers.

Standard torsemide was obtained from Macleods Pharmaceuticals Ltd. Acetonitrile (HPLC grade), was purchased from Qualigens, Mumbai. Potassium dihydrogen phosphate, ortho phosphoric acid, perchloric acid (all analytical reagent grade) were purchased from S. D. Fine Chem. Ltd., Mumbai. Pooled human plasma was purchased from the National Plasma Fractionation Center, K.E.M. Hospital Mumbai, and was stored at -20° until used.

Stock solution of torsemide were prepared at 1 mg/ml in methanol and stored at 4°. The stock solution of torsemide was further diluted with methanol to give series of standard solutions with concentration of 100, 250, 500, 1000, 2000, 3000, 4000 ng/ml. A standard curve consisting of seven points ranging from 100 to 4000 ng/ml and quality control samples consisting of lowest (100 ng/ml) median (1000 ng/ml) and highest (4000 ng/ml) were prepared by spiking appropriate amount of standard solution in blank plasma and stored at -20° until analysed.

The HPLC system consisted of a Jasco–PU980 intelligent pump (Jasco Ltd., Japan), manual injector port with 50 μl loop (Rheodyne, USA) and Jasco UV/Vis 975 intelligent detector (Jasco Ltd., Japan). The wavelength of the detector was set at 290 nm. Detector output was quantified on Jasco face (Version 2.0). The samples were separated by chromatography on a C18 column (HiQ Sil^®^, 4.6 mmφ × 250 mm), using acetonitrile and potassium dihydrogen orthophosphate buffer (0.05 M) in proportion of 40:60 (pH 4.0), respectively as a mobile phase, at a flow rate of 1 ml/min.

One millilitre of drug free blank plasma spiked with torsemide, 0.8 ml of 10% perchloric acid in methanol was added and vortexed for 30 s. The mixture was centrifuged at 10500 × g for 10 min. The supernatant (20 μl) was injected in the column.

Validation was carried out as per CDER guidelines (http://www.fda.gov/cder/guidance/index.htm accessed on Dec 2003). The developed method was validated for selectivity, limit of detection and quantitation, linearity, precision and accuracy, recovery, stability in plasma, system suitability and robustness. Quality control samples were used for inter-day and intra-day precision and accuracy of the assay. Spiked concentrations and peak area of torsemide were fit by linear regression equation (y=mx+c). Precision is reported as % CV of the estimated concentrations and accuracy expressed as [(mean calculated concentration/spiked concentration)×100]

The method described in this paper was applied to a bioequivalence study of two oral formulation of torsemide 20 mg tablet, Macleods Pharmaceutical Ltd., Mumbai (Test formulation) versus that of Dytor, Cipla Ltd., Mumbai (Reference formulation). Institutional Ethics Committee of Bombay College of Pharmacy approved the study protocol. Twelve healthy male Indian subjects were included in the study. The study was 12×2 single dose, randomized, open, and crossover design. Subjects were fasted overnight before drug administration. Five millilitre of blood samples were collected at 30, 45, 60, 75, 90 and 120 min and thereafter at 3, 4, 8, 12 and 24 h post drug administration through an indwelling cannula into heparinised tubes. The blood samples were immediately centrifuged, plasma was separated and stored at -20° until analysed. The plasma samples obtained at various time intervals were analysed by HPLC method developed. Pharmacokinetic parameters (C_max_, T_max_, T_1/2_, K_ele_, AUC_0-t_ and AUC_0-inf_) were calculated by Basica^®^ (V 1.12).

The purpose of the present paper was to develop a simple, rapid with simple sample preparation and simple mobile phase. Protein precipitation by perchloric acid gave no endogenous interference (selective) from plasma at the retention of the drug but recovery was low; while precipitation with methanol gave good recovery but the method was not selective. Hence, we tried a mixture of perchloric acid and methanol not only to get better selectivity as well good recovery of the drug. The mixture of perchloric acid and methanol was later optimized to 0.8 ml of 10% perchloric acid in methanol.

The selectivity of the method was evaluated by analysis of drug-free human blank plasma, which did not show any interference at the retention time of torsemide i.e. 5.00±0.20 min. The tailing factor of the torsemide peak is not greater than 1.5. The method was linear in the range of 100 - 4000 ng/ml. The regression coefficient ‘r’ was 0.999. The limit of detection and quantification was 50 ng/ml and 100 ng/ml, respectively. Intra day precision and accuracy was determined by analyzing quality control samples (100, 1000 and 4000 ng/ml) in no fixed order. Inter day precision was determined from quality control samples once each on five different days. The precision of the assay calculated as % CV and was less than 8.37% at all concentration studied. The accuracy of the assay was found in the range of 88.32 to 107.01% (Table 1). The percent recovery (n=5) was found to be more than 82% at all concentrations. Stock and working solution of torsemide in methanol were stable for at least two months when stored at 4°. The plasma control of torsemide also found to stable to three freeze-thaw cycles. The plasma controls stored in polypropylene tube stored at –20° were stable for at least 30 d. A relative standard deviation, calculated for five replicate injections of standard preparation is not more than 2.0%. The method showed good ruggedness in fact, little change in either mobile phase ratio or in normal laboratory condition of humidity, light, air exposure and temperature did not influence the retention time of torsemide.

**TABLE 1:** PRECISION AND ACCURACY OF QUALITY CONTROL STANDARDS OF TORSEMIDE

Spiked Conc. (ng/ml)	Intra day			Inter day		
		
	Mean Concentration±SD (ng/ml)	Accuracy	%CV	Mean Concentration±SD (ng/ml)	Accuracy	%CV
100	89.54±5.02	89.54	5.60	88.32±7.40	88.32	8.37
1000	1070.12±85.67	107.01	8.00	955.35±40.64	95.53	4.25
4000	4118.0±203.9	102.95	4.95	4176.13±135.93	104.40	3.25

The table gives mean percent recovery, calculated from five samples at each of the concentrations mentioned (n=5) along with their coefficient of variation (%CV).% Accuracy = [Measured concentration / spiked concentration]×100.

The mean peak plasma concentrations for 20 mg torsemide tablet were found to be 3175.9±428.9 ng/ml and 3399.4±581.5 ng/ml for the reference and test formulations respectively. These values were achieved at 1.0±0.4 and 0.8±0.2 h, respectively, for reference and test formulations. AUC_0-24_ was found to be 8123.3±2628.6 ng.h/ml and 8204.6±3447 ng.h/ml for the reference and test formulations respectively (Table 2). The bioavailability of the test formulation as judged from AUC_0-24_ was found to be 101.0% as compared to the reference formulation for torsemide. The 90% confidence limits for the ratio of the log normal transformed data of C_max_, AUC_0-24_, and AUC_0-inf_ of test product and reference product were found to in the range of 98.6 to 102.8, 101.8 to 105.3 and 102.4 to 105.5 and are within prescribed limits (80 to 125) (Table 2). Test formulation was found to be bioequivalent with the reference formulation

**TABLE 2:** PHARMACOKINETIC PARAMETERS OF TORSEMIDE

Parameters	Test	Reference	90%φ (80-125%)	*P* Valueψ
C_max_ (ng/ml)	3399.4±581.5	3175.9±428.9	98.6 to 102.8	0.21
T_max_ (h)	0.8±0.2	1.0±0.4		0.10
T_1/2_ (h)	3.8±2.6	3.2±1.7		0.28
K_ele_	0.24±0.1	0.27±0.1		0.29
AUC_0-t_ (ng.h/ml)	8204.6±3447	8123.3±2628.6	101.8 to 105.3	0.92
AUC_0-inf_ (ng.h/ml)	9035.0±4195.7	9061.7±3552.8	102.4 to 105.5	0.97

The table gives mean±standard error for comparative pharmacokinetic data of torsemide 20 mg tablet in 12 healthy volunteers along with the 90% confidence interval for log transformed data of C_max_ and AUC. The table also gives the p value, applied on log-transformed data (n=12).

φ Statistics were applied on logarithm transformed data, n=12.

ψ Non-significant difference at 95% confidence limits.

The method described in this paper is simple and rapid HPLC-UV method for determination of torsemide in human plasma and found to be precise and accurate; the proposed method was successfully applied for pharmacokinetic studies.
